# A large-scale comparative study of isoform expressions measured on four platforms

**DOI:** 10.1186/s12864-020-6643-8

**Published:** 2020-03-30

**Authors:** Wei Zhang, Raphael Petegrosso, Jae-Woong Chang, Jiao Sun, Jeongsik Yong, Jeremy Chien, Rui Kuang

**Affiliations:** 1 Department of Computer Science, University of Central Florida, 4000 Central Florida Blvd, Orlando, 32816 FL USA; 2 Department of Computer Science and Engineering, University of Minnesota Twin Cities, 200 Union Street SE, Minneapolis, 55455 MN USA; 3 Department of Biochemistry, Molecular Biology and Biophysics, University of Minnesota Twin Cities, 200 Union Street SE, Minneapolis, 55455 MN USA; 4 Department of Biochemistry and Molecular Medicine, University of California, Davis, 2700 Stockton Blvd., Sacramento, 95817 CA USA

**Keywords:** Isoform-level expression, Cross-platform comparison, NanoString, RNA-seq, Exon-array, Microarray gene expression

## Abstract

**Background:**

Most eukaryotic genes produce different transcripts of multiple isoforms by inclusion or exclusion of particular exons. The isoforms of a gene often play diverse functional roles, and thus it is necessary to accurately measure isoform expressions as well as gene expressions. While previous studies have demonstrated the strong agreement between mRNA sequencing (RNA-seq) and array-based gene and/or isoform quantification platforms (Microarray gene expression and Exon-array), the more recently developed NanoString platform has not been systematically evaluated and compared, especially in large-scale studies across different cancer domains.

**Results:**

In this paper, we present a large-scale comparative study among RNA-seq, NanoString, array-based, and RT-qPCR platforms using 46 cancer cell lines across different cancer types. The goal is to understand and evaluate the calibers of the platforms for measuring gene and isoform expressions in cancer studies. We first performed NanoString experiments on 59 cancer cell lines with 404 custom-designed probes for measuring the expressions of 478 isoforms in 155 genes, and additional RT-qPCR experiments for a subset of the measured isoforms in 13 cell lines. We then combined the data with the matched RNA-seq, Exon-array, and Microarray data of 46 of the 59 cell lines for the comparative analysis.

**Conclusion:**

In the comparisons of the platforms for measuring the expressions at both isoform and gene levels, we found that (1) the agreement on isoform expressions is lower than the agreement on gene expressions across the four platforms; (2) NanoString and Exon-array are not consistent on isoform quantification even though both techniques are based on hybridization reactions; (3) RT-qPCR experiments are more consistent with RNA-seq and Exon-array than NanoString in isoform quantification; (4) different RNA-seq isoform quantification methods show varying estimation results, and among the methods, Net-RSTQ and eXpress are more consistent across the platforms; and (5) RNA-seq has the best overall consistency with the other platforms on gene expression quantification.

## Background

In eukaryote genome, a single gene can contain multiple exons and introns, where the exons can be alternatively spliced together in different ways. Recent studies have estimated that alternative splicing events exist in more than 95% of multi-exon genes in human and mouse [1, 2], and the mechanism provides the opportunity to create protein isoforms of differing functions from a single gene in a cellular system. Therefore, elucidating gene expression at the isoform resolution could improve our understanding of molecular mechanisms and potentially improve molecular signals for cancer phenotype predictions [3, 4]. However, accurately quantifying isoform expression is significantly more challenging than estimating aggregated gene expressions due to the ambiguity in overlapped regions between alternative isoforms in the same gene [5].

**Fig. 1:**
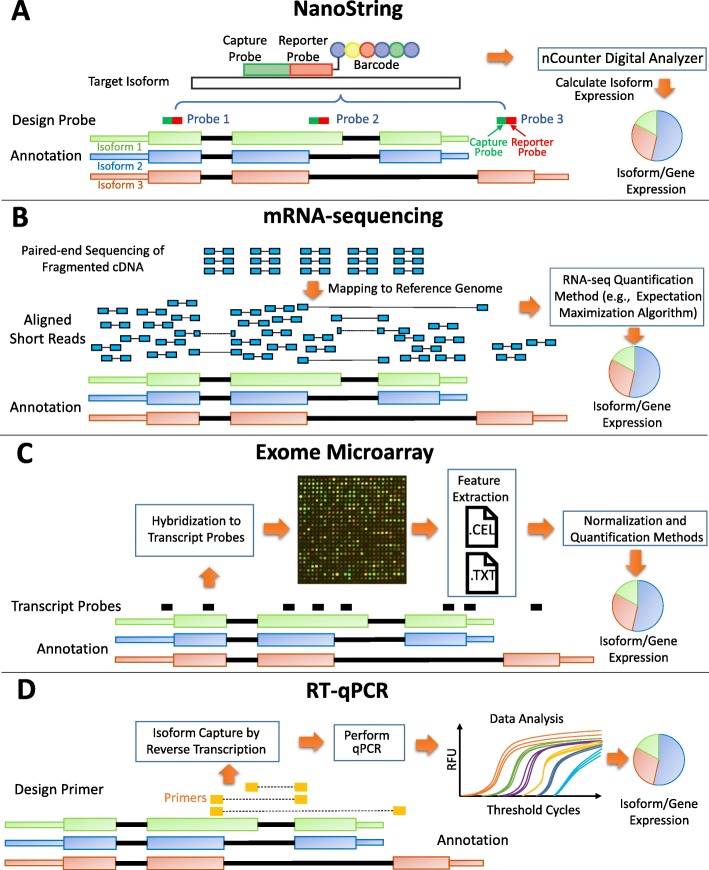
Four platforms. **a** NanoString nCounter. A capture probe works together with a reporter probe to capture the signal in the target sequences. Each capture-reporter probe pair is tagged with a distinct color-coded barcode to represent the detection of a single target molecule for direct digital readout. nCounter Digital Analyzer reports the expression levels of targeted mRNAs. In this example, Probe 1 measures the total expressions of all the isoforms of the gene; Probe 2 measures the expression of Isoform 1; Probe 3 measures the expression of Isoform 3. **b** mRNA-sequencing (RNA-seq). Paired-end sequencing of fragmented cDNAs generated by next-generation sequencing technology are aligned to the reference genome. The read coverage can be analyzed to infer the mRNA abundance by RNA-seq quantification methods. **c** Exome Microarray. In the Microarray experiment, exon-level expressions are estimated based on the hybridization intensity measurements by multiple probes targeting the putative exonic regions. After normalization, quantification methods are applied to estimate gene and isoform expressions. **d** Quantitative reverse transcription PCR (RT-qPCR). In this platform, RNAs are first transcribed into cDNAs by reverse transcriptase from the total RNA. Then the cDNAs are used as the template for the qPCR reaction. The gene and isoform expression can be estimated based on the amplified DNAs after performing qPCR

Several high-throughput platforms have been developed during the last decades for transcriptome studies, including mRNA sequencing (RNA-seq), array-based technologies (Microarray and Exon-arrays), quantitative reverse transcription polymerase chain reaction (RT-qPCR), and the more recently developed NanoString’s nCounter technology (Fig. 1). Currently RNA-seq is the most commonly used platform for measuring the expressions in a transcriptome. RNA-seq provides more sensitive measuring of gene and isoform expressions, and detects both known and novel features in a single assay (e.g., transcript isoforms, gene fusion, single nucleotide variants) without requiring prior knowledge compared to array-based technologies [5–7]. While at a lower-throughput, RT-qPCR enables the determination of the exact amounts of amplified DNAs in a sample, which are often considered as the gold standard to confirm the results of the studies based on RNA-seq or Microarray. The NanoString nCounter platform captures and counts individual mRNA transcripts. Its advantages over the other platforms include direct measurement of mRNA expression levels without enzymatic reactions, high sensitivity coupled with high multiplex capability, and digital readout [8, 9]. The strengths and limitations associated with each platform are described in Table 1. Due to these discrepancies, different platforms can report inconsistent isoform expressions measured on the RNAs extracted from the same cell sample.

**Table 1:** Advantages and limitations of each platform

	**Advantages**	**Limitations**
**NanoString**	(1) RNAs are directly measured without amplification or cloning; (2) Digital readout generates less background noise; and (3) Allows high dynamic range of expressions.	(1) Only a limited number of probes are available to measure isoform or gene expression; and (2) Sophisticated custom probe design is often needed.
**RNA-seq**	(1) No requirement of gene annotations; (2) Capable of detecting novel splicing isoforms; (3) Allows high dynamic range of expressions; and (4) Provides transcriptome-wide expression profiling.	(1) Transcript specific bias/ 3’-end bias; and (2) Various sampling biases as a result of library preparation protocols.
**Exon-array**	(1) Provides transcriptome-wide expression profiling; and (2) Capable of measuring expressions at exons and exon-junctions.	(1) Limited dynamic range; (2) Requires a higher amount of molecules in RNA preparation; and (3) Depends on existing genome annotation.
**RT-qPCR**	(1) High dynamic range of expressions; (2) Less biased results; and (3) much cheaper in comparison to RNA-seq and Microarray on a small-scale study.	(1) Only a limited number of transcripts can be measured; and (2) Custom primer designs are often needed.

In this paper, we study the correlations of the expressions estimated by four different platforms including RNA-seq, NanoString nCounter, Microarray/Exon-array and RT-qPCR, at isoform and gene levels to better understand the characteristics of the estimations made by each platform. To assess the correlations across the platforms, we first performed custom NanoString nCounter experiments on 59 cancer cell lines with 404 custom-designed probes for measuring the expressions of 478 isoforms in 155 genes. We then combined the nCounter data with the matched RNA-seq, Exon-array and Microarray data of 46 of the 59 cell lines from Cancer Cell Line Encyclopedia (CCLE) [10] and Gene Expression Omnibus (GEO) [11] to perform isoform and gene abundance estimation on raw expression profiles of the 46 cell lines on all the platforms. Additional RT-qPCR experiments for a subset of the measured isoforms in 13 cell lines were also performed for validations.

Previous studies have demonstrated the agreement between RNA-seq and array-based platforms for isoform expression estimation [5] and gene expression estimation [6, 12, 13]. However, none of the studies has included large-scale NanoString data at isoform level in the comparison, nor did the studies investigate large-scale isoform profiles across multiple cancer types. Better understandings of the characteristics of the gene and isoform expression estimations with the four platforms could lead to better strategies to design experiments for cancer studies, and integrate different platforms together to more accurately estimate the mRNA expressions for applications in disease biomarker detection, cancer outcome prediction, drug target identification, and drug response prediction.

## Results

### Cancer cell line data preparation

The NanoString experiment was conducted on 59 cell lines, including 12 ovary cell lines, 12 lung cell lines, 11 colon cell lines, 10 breast cell lines, four pancreas cell lines, two prostate cell lines, two stomach cell lines, and six cell lines of six other types of tissues as listed in Table S1 in the supplementary document. A total of 404 probes were customized to estimate the isoform expressions of 155 cancer genes curated from the literature [14] with more reliable isoform annotations, where each of the 155 genes contains at least two isoforms. As shown in Table S1, 46 out of the 59 cell lines are also the cancer cells in CCLE. The raw mRNA-Sequencing (RNA-seq) data and Microarray expression data (HG-U133 Plus 2.0 Array) of the 46 cancer cell lines were downloaded from CCLE and processed for comparison with the NanoString nCounter data. Additionally, the raw Microrarray expression data of the same 46 cancer cell lines were downloaded from GEO as the replicates of the CCLE Microarray data in the analysis. The raw Exon-array (HuEx.1.0.st.v2) data of 35 of the 59 cancer cell lines were also downloaded from GEO to compare with the other platforms. Finally, an RT-qPCR experiment was designed to measure the expressions of a subset of the genes and isoforms in 13 cell lines to validate the expressions measured on the other platforms. The complete list of the cell lines used in this study and the data availability on each platform is available in Table 2 and Table S1.

**Table 2:** Summary of the availability of cell line data in each platform

	**# of cell lines**	**# of genes**	**# of isoforms**	**Resource**	**Platform**
**NanoString**	59	155	478		nCounter
**RNA-seq**	46	transcriptome-wide	transcriptome-wide	CCLE	Illumina HiSeq 2000
**Microarray gene expression**	46	transcriptome-wide	transcriptome-wide	CCLE and GEO	HG-U133 Plus 2.0 Array
**Exon-array**	35	transcriptome-wide	transcriptome-wide	GEO	Affy HuEx.1.0.st.v2
**RT-qPCR**	13	4	8		

### Comparison of isoform expression profiles across platforms

#### Low consistency among the estimated isoform expressions across the platforms.

**Fig. 2:**
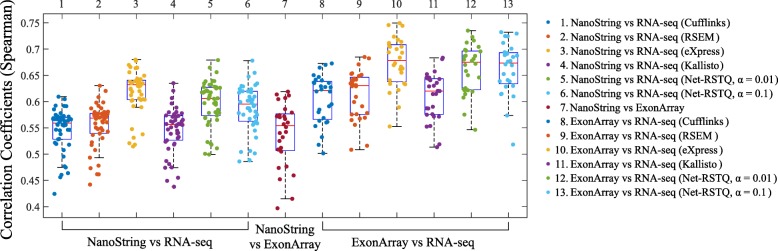
Correlations between estimated isoform expressions on two platforms. In the boxplots, each dot represents the correlation coefficient measured on one compared cell line. Five different isoform quantification methods are used to generate the profiles with RNA-seq data in the comparison

The correlations of isoform expressions among different platforms are reported in Fig. 2. Each dot in the boxplot represents the Spearman correlation coefficient of the 478 isoforms expressions in 155 genes between two platforms for one cell line. Each boxplot shows the correlation coefficients measured for all the cell lines between two compared platforms. Five different isoform quantification methods (Net-RSTQ [3], Cufflinks [15], RSEM [16], eXpress [17] and Kallisto [18]) were used to quantify the isoform expressions for the RNA-seq data.

**Fig. 3:**
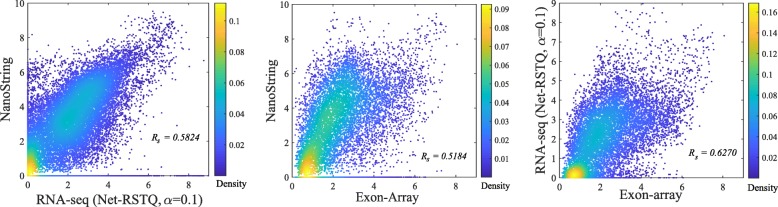
Scatter plots of estimated isoform expressions between two different platforms. Each dot represents one isoform in one cell line. The isoform quantification by Net-RSTQ is applied to the RNA-seq data in the comparison. The overall correlation is also reported for each comparison

In general, the Spearman correlations of isoform expressions between NanoString and RNA-seq (median ***R***_***s***_=0.55∼0.63) are lower than the correlations between Exon-array and RNA-seq (median ***R***_***s***_=0.62∼0.68). The correlations between the NanoString and Exon-array are relatively low (median ***R***_***s***_=0.55). Figure 3 shows the overall correlation coefficients between two platforms with isoform quantification method Net-RSTQ for RNA-Seq. Each dot in the scatter plots represents one isoform in one cell line. The overall correlation coefficients are between 0.52 and 0.63 in the three comparisons. In a similar analysis on gene-level expression later reported in Figs. 6 and 7, the overall correlations are much higher (median ***R***_***s***_=0.68∼0.82). The observation is consistent with the fact that besides mapping reads to genes, additional analysis steps are required to resolve the read assignment to the shared exon region(s) for estimating the isoform expressions, and thus, compared to gene expression quantification, the consistency across the platforms on the estimated isoform expressions is lower.

#### NanoString and Exon-array are less consistent on isoform quantification

**Fig. 4:**
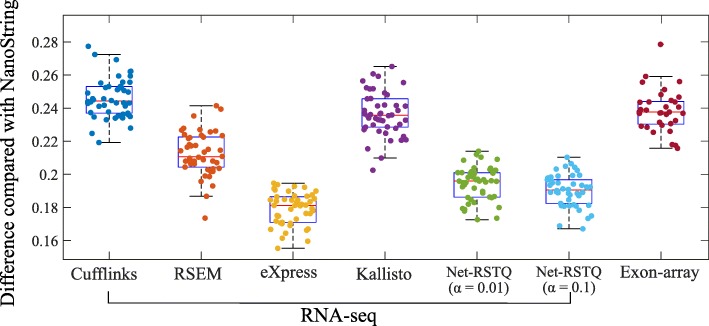
Validation by comparison with NanoString. The boxplots show the differences between NanoString and the other two platforms (RNA-seq and Exon-array) on isoform proportion estimation. Each dot represents the average isoform proportion difference between NanoString and one quantification method for RNA-seq, or Exon-array, in one cell line

It is also beneficial to measure the isoform proportions in each gene rather than the exact isoform expressions. For example, an investigator might only need to know which isoform has higher expression than the other isoforms in the gene. In Fig. 4, the isoform proportions estimated by NanoString were used as the reference proportions to evaluate different RNA-seq quantification methods and quantification based on the Exon-array platform. The differences of isoform proportions estimated by NanoString and the other platforms are measured by Eq. 2 in the “Methods” section. As shown in Fig. 4, the isoform proportions estimated by Exon-array are less consistent with the proportions estimated by NanoString, compared to those estimated by most RNA-seq quantification methods, even though the hybridization reaction is a key step in both nCounter and array-based experiments but not RNA sequencing. This observation in this isoform proportion comparison also is consistent with the results for isoform expression comparison in Figs. 2 and 3.

#### Net-RSTQ and eXpress provide more consistent isoform quantification with RNA-seq data

In both Figs. 2 and 4, the compared isoform quantification methods on RNA-seq data show different levels of consistency with the other two platforms. In Fig. 2, the rankings of median correlations for the five methods are similar in the comparisons with NanoString and Exon-array platforms, even if the quantification results are not consistent between NanoString and Exon-array. Specifically, eXpress [17] and Net-RSTQ [3] are the most consistent methods, followed by RSEM [16], Kallisto [18] and Cufflinks [15]. Evaluated by the NanoString data and Exon-array data, eXpress and Net-RSTQ seem to provide more reliable isoform quantifications with RNA-seq data compared with the other methods. Similar results are also observed in the isoform proportion analysis in Fig. 4. Net-RSTQ and eXpress provide the most consistent estimations of proportions compared with the estimation on the NanoString data.

#### RT-qPCR experiments agree more with the RNA-seq and exon-array results

Among the genes that NanoString, RNA-seq and Exon-array report the most different quantification results for, we selected four genes (ASXL1, LRIG3, NOTCH2 and SF3B) to perform RT-qPCR experiments in the 13 cell lines with relatively high expression levels and feasibility of designing isoform-specific primers.

**Fig. 5:**
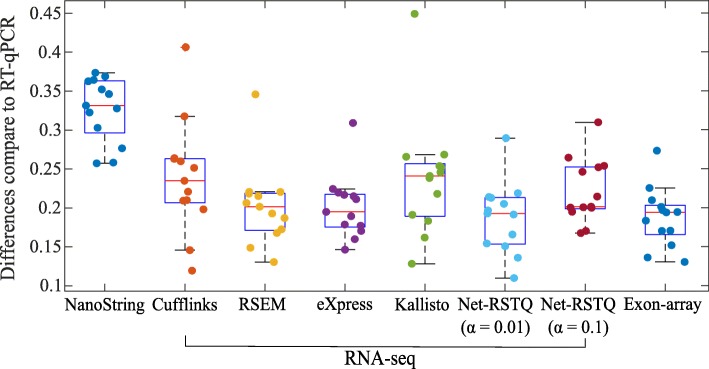
Validation with RT-qPCR results. The boxplots show the differences in the estimation of isoform proportions between RT-qPCR and the other platforms. Each dot represents the average isoform proportion differences between RT-qPCR and another platform or one quantification method on RNA-seq data in one cell line

The isoform proportions estimated by NanoString data, Exon-array data, and five different methods for RNA-seq data analysis were compared to the RT-qPCR estimation in the boxplots in Fig. 5 and scatter plots in Figure S2 in the supplementary document. In general, the isoform proportions estimated by RNA-seq and Exon-array show better agreement with RT-qPCR quantification than NanoString. The median differences (Eq. 2 in the “Methods” section) across 13 cell lines are between 0.20 ∼0.24 for RNA-seq platform and 0.20 for Exon-array platform, while the median difference is 0.33 in comparison to NanoString, which is significantly higher than RNA-seq and Exon-array (*p*-values < 1e-3 by The Wilcoxon Rank-Sum Test).

**Fig. 6:**
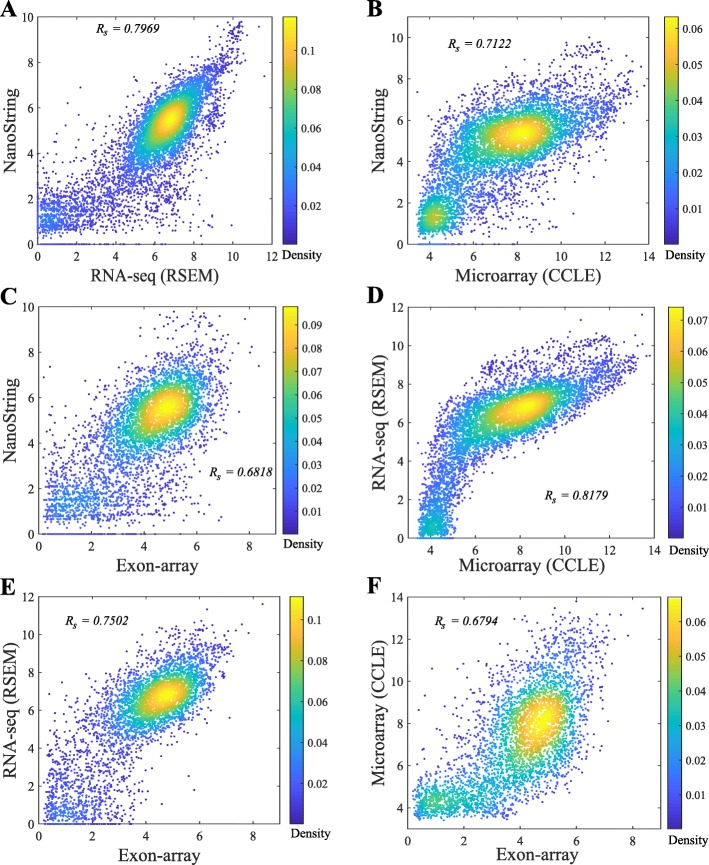
Scatter plots of gene expressions measured on two platforms. Each dot represents one gene in one cell line. RSEM is applied for RNA-seq quantification and the CCLE Microarray gene expression data are used in the comparison. The overall correlation is also reported for each comparison

**Fig. 7:**
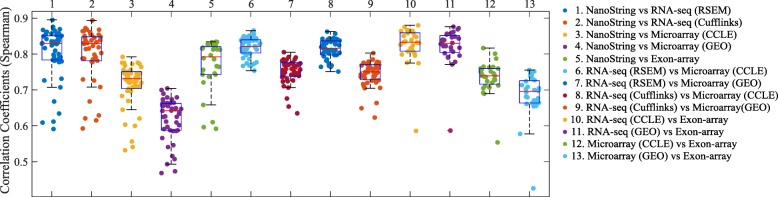
Correlation between gene expressions measured on two platforms. The box plots show the results of different cancer cell lines with each dot representing the correlation coefficient for one cell line. Two different RNA-seq quantification methods and two different versions of Microarray gene expression datasets (CCLE and GEO) are compared

### Comparison of gene expression profiles across platforms

#### RNA-seq is consistent with other platforms on gene expression quantification.

The scatter plots comparing the gene expressions estimated by four platforms are shown in Fig. 6. RSEM was applied to RNA-seq quantification and CCLE Microarray data was used in this comparison. The correlations on the gene expressions between RNA-seq and the other three platforms shown in Fig. 6a, d and e are the most consistent in the comparisons of each pair of platforms (***R***_***s***_=0.75∼0.82). The other three paired comparisons on the other three platforms shown in Fig. 6b, c and f show correlation coefficients between 0.68 and 0.71. Although Microarray and Exon-array platforms were both array based, the correlation was the lowest in all the six pairs. The high consistency between Microarray and RNA-seq platforms may have benefited from the same experimental conditions and cell materials used in the CCLE project. A more comprehensive comparison is shown in Fig. 7. Each dot in the boxplots represents the correlation coefficient of the expressions of 155 genes in one cell line between two platforms. Even though the gene expressions in the cell lines were estimated by the same platform in the Microarray (GEO) and Microarray (CCLE), the correlation is not as high as expected (Figure S6 in the supplementary document). In Fig. 7, Cufflinks and RSEM yield similar consistent gene expressions with the other platforms in the RNA-seq data.

#### NanoString, RNA-seq and Exon-array detect lowly expressed genes with less consistency

Similar to the observations in previous studies[7], the results in Fig. 6 also show that Microarray platforms are not sensitive enough to detect lowly expressed genes due to the background noise from cross-hybridization. There is a clear pattern that in the range of lowly expressed genes estimated by NanoString and RNA-seq data, Microarray gene expressions are all at the lowest end with very small differences. Exon-array assigns many probes to the exons in a gene and thus, is slightly more sensitive than the Microarray that only measures gene expressions.

**Fig. 8:**
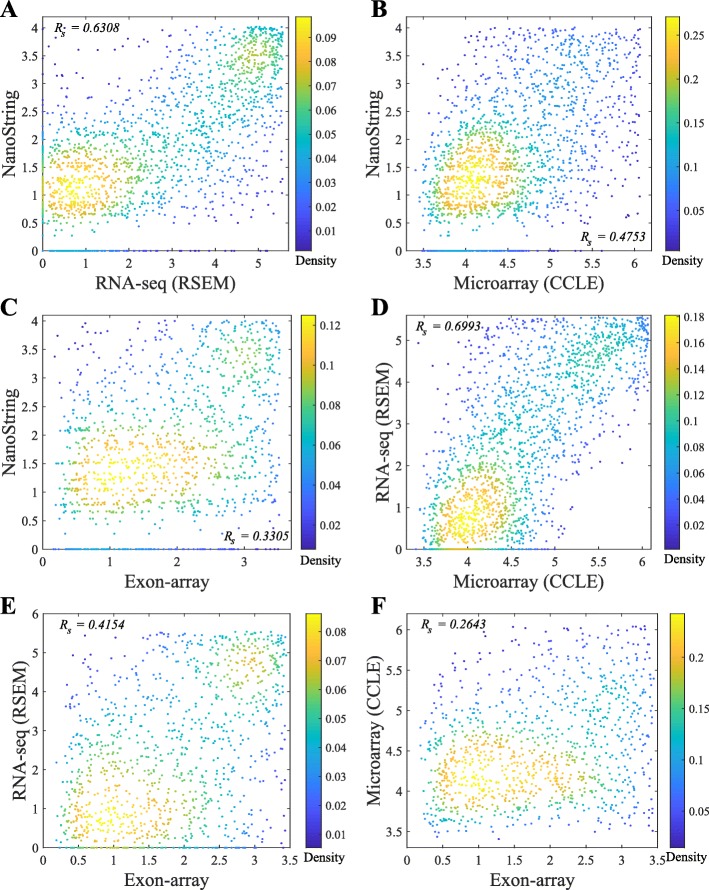
Scatter plots of the lowly expressed genes measured on two platforms. Each dot represents one gene in one cell line. RSEM is applied for RNA-seq quantification and the CCLE Microarray gene expression data is used in the comparison

The pairwise comparison of lowly expressed genes across the platforms in Fig. 8 also indicates that the detected expressions of the low-abundance genes are less consistent across NanoString, RNA-seq, Exon-array, and Microarray. Some of the genes were very rarely expressed in one platform, but highly expressed in the other platform(s). Across the four platforms, Exon-array has the least agreement with the other three platforms.

#### Fold change and differential expression analysis

**Fig. 9:**
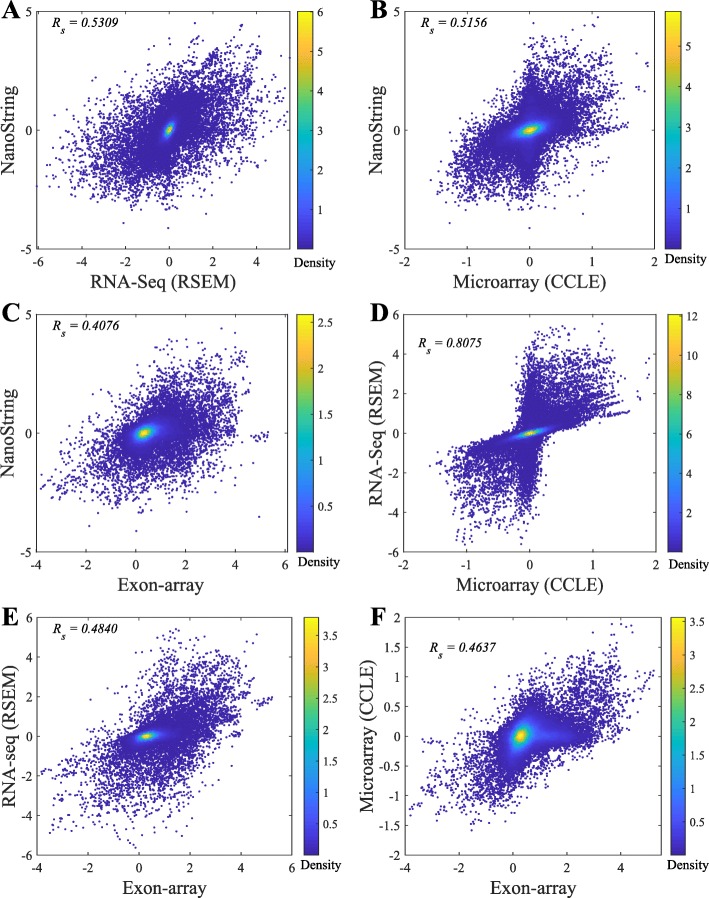
Scatter plots of fold-changes in gene expressions (breast cancer cell line versus another cell line) between two platforms. Each dot represents the fold-change of one gene between each breast cancer cell line and one cell line of the other cancers. RSEM is applied for RNA-seq quantification and the CCLE Microarray gene expression data is used in the comparison

The fold-change was calculated based on breast cancer cell lines versus all the other cell lines. The correlations of fold-change between platforms are reported in Fig. 9. Compared to the gene-expression correlations in Fig. 6, the agreement in fold changes is lower. Among all the six pairwise comparisons, RNA-seq and Microarray show the highest consistency on the fold-change analysis in Fig. 9d. The “x-shape” in Fig. 9d shows that the up/down regulated genes detected by RNA-seq and Microarray platforms are consistent. However, in all the other plots in Fig. 9, there are always some genes show opposite regulations in the two compared platforms. In general, there is an agreement between the correlation in gene expressions in Fig. 6 and the correlation in fold changes in Fig. 9.

In the differential gene expression analysis, ANOVA was applied to detect the top differentially expressed genes across six tissue types (ovary, lung, colon, breast pancreas, and prostate) on the gene expressions estimated by NanoString, RNA-seq and Microarray. The top-10 genes identified from each platform are reported in Table 3 and Figure S3-S5 in the supplementary document. Only MYB is identified by all three platforms. MYB and EWSR1 are the only common genes detected by both NanoString and Microarray. Six out of the top-10 genes identified from RNA-seq were also detected either by NanoString or Microarray. The complete lists of the differentially expressed genes identified from three platforms are reported in Table S2 in the supplementary document.

**Table 3:** The top-10 differentially expressed genes

**NanoString**		**RNA-seq**		**Microarray**	
**Gene name**	***p-value***	**Gene name**	***p-value***	**Gene name**	***p-value***
MYB**	7.176e-06	LIFR*	3.813e-05	MYB**	1.132e-05
LIFR*	1.667e-04	MYB**	6.103e-05	PRDM16	2.846e-03
ATRX	8.292e-04	PRF1	1.359e-04	SLC34A2	3.766e-03
SMARCA4	3.455e-03	SLC34A2*	5.697e-04	EML4*	4.375e-03
KLK2	8.929e-03	ZNF331	8.192e-04	HNRNPA2B1	4.783e-03
ETV4*	0.01022	WWTR1	2.557e-04	SYK	5.649e-03
EWSR1*	0.01138	EML4*	3.915e-03	EWSR1*	5.877e-03
MSI2	0.01167	ETV4*	7.149e-03	BCL6*	8.001e-03
SRSF2	0.01218	TFG	8.614e-03	NONO	8.039e-03
TCF3	0.01561	BCL6*	9.211e-03	GPC3	8.258e-03

**The genes identified by three platforms. *The genes identified by two platforms

## Discussion

During the last decade, mRNA sequencing has gradually taken over transcriptome-wide gene expression profiling from array-based technologies. NanoString nCounter platform promises to yield less biased and more scalable experimental results compared to RT-qPCR for targeted (customized) expression profiling. Many researchers and medical practitioners choose one platform over another without knowing the expected differences in the outcomes of the technologies. In this study, we evaluated four different platforms on both isoform and gene expression profiling for a large number of cancer cell lines. The experiments showed relatively high agreement among the platforms on gene expression profiling (Fig. 6), while isoform expressions estimated by the platforms were less consistent (Fig. 2). Collectively, the results suggest isoform quantification is still a more challenging problem than gene expression profiling. Both the experimental platform and the isoform quantification method play a critical role for reliable estimation of isoform expressions. Potentially, integration of the isoform expression data collected across the platforms may improve the reliability of isoform expression estimation.

A major challenge of evaluating isoform expression quantification techniques is the lack of large-scale benchmark data with ground-truth isoform expression levels. RT-qPCR is usually considered as the gold-standard for validating the results from the analysis of RNA-seq and array-based data [5, 13]. While in our experiments, NanoString platform did not show more consistent results with array-based techniques and RT-qPCR compared with RNA-seq (Figs. 2, 3, 4, 5, 6, 7, 8 and 9), the discrepancy might be inherent between NanoString and the other three platforms—nCounter technology does not rely on amplification and reverse transcription as RNA-Seq, RT-qPCR and Microarray technologies do. RNA is commonly converted to more stable complementary DNA (cDNA) by reverse transcription for PCR amplification. It is desirable that the resulting cDNA population represents the original RNA population. However, both reverse transcription and PCR amplification introduce biases based on the base-compositions of gene transcripts in the quantification of the transcripts. The high error rate of reverse transcription can also impact data quality. In addition, the current technologies require that the cDNA molecules represent only fragments of the RNAs in an appropriate size for sequencing or hybridization to Microarray. The fragmentation sites are often non-random as a result of sequence-dependent features such as GC-content, epigenomic modifications and DNA conformational energy. Though the bias can be traced back to specifics of the preparation protocols, it is not possible to predict fragment distribution directly from a protocol in different technologies. Therefore, it is not clear if NanoString is indeed less reliable than RNA-seq. In principle, the NanoString nCounter technique holds several advantages over RT-qPCR and RNA-seq, such as directly measuring RNAs without amplification or cloning, no enzymatic steps required, high level of sensitivity since both the probes and the target are in solution rather than being bound to a surface, and digital readout. The current limit of nCounter is to measure up to 800 probes in one experiment, which is far from sufficient for transcriptome-wide profiling. Moreover, the capture and reporter probes can only be designed to identify known isoforms, and thus NanoString technology is not capable of detecting novel transcripts. In addition, it is not guaranteed that the isoforms in one gene can be completely distinguished by probe design, and therefore, isoform quantification is only feasible to a subset of the genes. For this reason, this study only focuses on 478 transcripts in 155 cancer genes, and the designed probes cannot distinguish every transcript in these genes. Due to this limitation, the conclusions in this study are made only based on this subset of genes.

## Conclusions

This study made several additional useful observations. First, Net-RSTQ and eXpress tend to produce more consistent isoform quantification on the RNA-seq data than the other compared isoform quantification methods. Different from the other methods, Net-RSTQ is a network-based approach that directly incorporates additional information from protein domain-domain interactions to overcome the limitations of short-read alignments for transcript quantification, since the protein products of highly co-expressed transcripts are more likely to interact with each other by protein domain-domain binding [3]. The eXpress model applies additional normalization strategies by jointly estimating parameters for the fragment-length distribution, sequencing errors and sequencing biases to improve the isoform expression estimation [17]. Second, NanoString and RNA-seq are more sensitive to lowly expressed genes than the array-based platforms, but the profiling by the two platforms are still not consistent with each other in the low-expression range. Third, the agreement among the platforms on fold-change analysis is moderate, which is lower than the agreement on gene expression analysis, suggesting that fold-change normalization against another sample will decrease the agreement across platforms. Fourth, the top differentially expressed genes across different tissue types identified by RNA-seq agree with the detection by one or both of the other platforms, while the other two platforms have less agreement. Fifth, the gene expression profiling results by the Microarray (GEO), in which the data were combined from the datasets generated by different labs in different studies, show lower agreement with the profiling by the other platforms than the profiling by Microarray (CCLE), in which the samples were generated in the same study (Fig. 7). This might be explained by the technical and experimental biases among the Microarray (GEO) samples prepared and sequenced by different laboratories. Further normalization to remove the biases might improve the consistency with the other platforms.

## Methods

### NanoString probe design and nCounter experiments

**Fig. 10:**
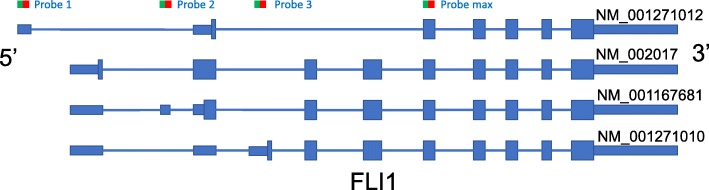
Probe design of gene FLI1. There are four isoforms in gene FLI1 and four probes were designed to distinguish the expressions of the isoforms. *Probe max* measures the expression for the gene, and the other three probes were designed to quantify three individual isoforms

We designed an experiment for measuring the isoform expressions of 155 multi-isoform cancer genes in 59 cell lines. The complete list of the 59 cell lines and their catalogue numbers are available in Table S1. Among the 155 genes, 79 genes contain two isoforms, 37 genes contain three isoforms, 18 genes contain four isoforms, and 21 genes contain more than four isoforms. We customized 404 capture-reporter probes to capture the 478 isoform expressions in the genes based on the RefSeq hg19 annotation (RefSeq release 66). In addition, 14 spike-in probes for quality control and 10 probes for measuring 10 house keeping genes for normalization of the data are also introduced. Each probe is 100 bps long, designed to capture a target sequence in one or multiple isoforms. For each gene, the probes were designed to distinguish the expressions of all known isoforms in the gene. In most of the genes, especially those with more than two isoforms, one of the probes was designed to measure the overall gene expression. We showed one real example in Fig. 10: four probes were designed to measure the four isoform expressions in gene FLI1. *Probe max* measures the expression of gene level. *Probes 1*, *2*, and *3* measure the isoform expression of NM_001271012, NM_001167681, and NM_001271010, respectively. The expression level of isoform NM_002017 can be calculated by taking the gene expression minus the total expression of the other three isoforms. Two technical replicates of each cell line were measured. The log(***x***+1) normalized probe signals for all 404 probes from two technical replicates are shown in Figure S1. The plot shows that the nCounter experiments are highly reproducible: a linear fit to the log transformed data results in a correlation coefficient of 0.9867. The dataset is deposited to GEO (GSE133226).

### NanoString gene and isoform abundance estimation

The geometric mean of the ten house keeping genes (TAF5L, HPS6, DNTTIP2, GNRHR, ZNF407, KCNK7, KIAA1539, RBM12, DGCR14, and KHDRBS1) was applied to normalize the NanoString signal in each cell line. Then, the expressions of isoforms in one gene can be estimated by minimizing the differences between the assessed and observed intensities for all probes in the gene as:

1 $$ \begin{aligned} & \underset{\boldsymbol{x}}{\text{min}} & & ||\boldsymbol{y}-{\boldsymbol{A}\boldsymbol{x}}||^{2}_{F}\\ & \text{s.t.} & & x_{i} \geq 0,\\ \end{aligned}  $$

where vector ***y*** represents the probe intensities. ***A*** is a *m*-*by*-*n* indicator matrix with values 0 or 1, where *m* is the total number of probes, and *n* is the total number of isoforms in the gene. If probe *j* covers the exon region of isoform *i* in the gene, then ***A***_(*j*,*i*)_=1, otherwise ***A***_(*j*,*i*)_=0. Vector ***x*** represents the isoform expressions to learn. After the isoform expression vector ***x*** is learned, the total gene expression can be derived by accumulating the isoform expressions. Finally, the gene and isoform expressions are all normalized by log(***x***+1).

### RNA-seq gene and isoform abundance estimation

The RNA-seq paired-end reads were aligned and quantified using hg19 reference genome with STAR [19] with default parameters. RNA-seq gene abundances were estimated using Cufflinks [15] and RSEM [16] and the isoform abundances were estimated using Net-RSTQ [3], Cufflinks, RSEM, eXpress [17], and Kallisto [18] with RefSeq annotation [20]. The Fragments Per Kilobase Million (FPKM) values were reported by Cufflinks and Transcripts Per Million (TPM) values were reported by RSEM, eXpress, Net-RSTQ, and Kallisto for each isoform (transcript) as the expression value. The gene-level expressions were estimated by summing all the isoform abundances in the gene. To account for the large dynamic range of abundances, the expressions are finally normalized by log(FPKM+1) or log(TPM+1).

### Exon-array gene and isoform abundance estimation

Gene and isoform expressions were generated from the raw Exon-array data (.CEL files) of the 35 cancer cell lines, using Multi-Mapping Bayesian Gene eXpression (MMBGX) [21]. Isoforms and genes were quantified using the Ensembl hg19 reference annotation (Ensembl release 70). The method disaggregates the signal between alternative transcripts of a gene to estimate the expression of each individual isoform. To compare Exon-array with other platforms, we only used the genes with the same annotations in both Ensembl and RefSeq annotations.

### Microarray gene abundance estimation

We collected two independent Microarray gene expression datasets for the same 46 cancer cell lines from the CCLE^1^ and GEO^2^ websites. The raw CEL files were normalized by Robust Multichip Average (RMA) [22]. After merging probes by gene symbols according to the RefSeq reference annotation, gene expressions derived from the 22,283 probes are included in this study.

### RT-qPCR experiment design

The RT-qPCR experiment was designed to measure the isoform proportion of four multi-isoform genes (ASXL1, LRIG3, NOTCH2 and SF3B) in 13 cell lines. The four genes were selected from the genes with the most different quantification results reported by NanoString, RNA-seq and Exon-array platforms, and based on the availability of the primers to distinguish the isoforms in the genes in the RT-qPCR experiments. The 13 human cell lines were selected based on the availability of cell culture in our labs.

In the experiments, RNAs from the cells were isolated by the Trizol method, according to the manufacturer’s protocol (Thermo Fisher Scientific^3^). Reverse transcription reaction using Oligo-d(T) priming and superscript III was carried out according to the manufacturer’s protocol. (Thermo Fisher Scientific^4^). The SYBR Green (Bio-Rad) was used to detect and quantify PCR products in real-time reactions. The comparative Ct method was used for data analyses, and we normalized the Ct values to the amount of total RNAs. The primer sequences used to measure the expression for each transcript are the following:hASXL1 iso1,2 for TAAACTGCCTGGCCGAATCAhASXL1 iso1 (NM_001164603.1) rev TAAGATGAAGGGGCCTGGTGhASXL1 iso2 (NM_015338.5) rev CTGTAGCTGGATGGCGAGAChLRIG3 iso1,2 rev CTCATGGAACTTGCCTTGATGAhLRIG3 iso1 (NM_001136051.2) for TTGTTCTCCCTCTGCTTGCThLRIG3 iso2 (NM_153377.4) for CGTCTTCCCGAGCCACTChNOTHC2 iso1,2 for ACCTTGTGAACCATTTCAAGTGChNOTHC2 iso1 (NM_024408.3) rev GGCACAGTCATCAATGTTCTCThNOTHC2 iso2 (NM_001200001.1) rev GACAATGCCCTGGATGGAAAAhSF3B iso1,2 for GGCGGACCATGATAATTTCCChSF3B iso1 (NM_012433.3) rev TTAGGATCAGGGGTTTTCCCTChSF3B iso2 (NM_001005526.2) rev TCAGCAGTTCTGACTTCAAGC.

**Cell culture**: Human cancer cell lines were obtained from the American Type Culture Collection (ATCC; www.atcc.org) and cultured according to standard mammalian tissue culture protocols and sterile technique:MCF7 (https://www.atcc.org/Products/All/HTB-22.aspx#culturemethod)AGC (https://www.atcc.org/products/all/CRL-1739.aspx#culturemethod)MDA-MB-231 (https://www.atcc.org/products/all/HTB-26.aspx#culturemethod)T-47D (https://www.atcc.org/products/all/HTB-133.aspx#culturemethod)HCT-15 (https://www.atcc.org/products/all/CCL-225.aspx#culturemethod)Hep G2 (https://www.atcc.org/products/all/HB-8065.aspx#culturemethod)HCT 116 (https://www.atcc.org/products/all/CCL-247.aspx#culturemethod)HT-29 (https://www.atcc.org/products/all/HTB-38.aspx#culturemethod)HT-1080 (https://www.atcc.org/products/all/CCL-121.aspx#culturemethod)PC-3 (https://www.atcc.org/products/all/CRL-1435.aspx#culturemethod)DU 145 (https://www.atcc.org/products/all/HTB-81.aspx#culturemethod)A549 (https://www.atcc.org/products/all/CCL-185.aspx#culturemethod)BT-549 (https://www.atcc.org/products/all/HTB-122.aspx#culturemethod).

### Compare isoform quantification consistency

The differences of isoform proportions estimated by two platforms are measured by the following formula:

2 $$ d = \frac{\sum_{i=1}^{n}\frac{\sum_{j=1}^{m_{i}}|p^{a}_{ij}-p^{b}_{ij}|}{m_{i}}}{n},  $$

where *i* is the index of the *n* genes in the cell line, *j* is the index of the *m*_*i*_ isoforms in the gene *i* in the cell line, *a* and *b* denote two different platforms, and $p^{a}_{ij}$ denotes the proportion of the *j*th isoform in the gene *i* in the platform *a*. For each gene *i*, $\sum _{j=1}^{m_{i}}p^{a}_{ij} = 1$.

### Correlation, fold-change and differential expression analysis

Cell line by cell line correlations of the isoform and gene expressions between two platforms were evaluated by the Spearman correlation coefficient. The lowly expressed genes were defined as the bottom one-third genes by expression in each platform. The intersection of the lowly expressed gene lists from two platforms were used for comparison.

The fold-change between the cell lines in one tissue type and all the other cell lines was calculated to compare the power of detecting the differentially expressed genes among the different platforms. RSEM for RNA-seq were used to derive the gene expressions for the differential expression analysis. The top differentially expressed genes were detected by ANalysis Of VAriance (ANOVA) [23] across different tissue types.

## Supplementary information

**Additional file 1** Figures S1-S6 and Table S1-S2.

## Data Availability

The raw NanoString data is deposited to GEO (GSE133226). All the source code and processed datasets in this study are available at: https://github.com/compbiolabucf/Cross-Platform-Comparison.

## Abbreviations

ANOVA: Analysis of variance
CCLE: Cancer cell line encyclopedia
cDNA: Complementary DNA
Ct: Cycle threshold
FPKM: Fragments per kilobase million
GC-content: Guanine-cytosine content
GEO: Gene expression omnibus
RefSeq: Reference sequence
RMA: Robust multichip average
RNA-seq: mRNA sequencing
RT-qPCR: Quantitative reverse transcription polymerase chain reaction
TPM: Transcripts per million

## Footnotes

1 https://portals.broadinstitute.org/ccle

2 https://www.ncbi.nlm.nih.gov/geo/

3 https://assets.thermofisher.com/TFS-Assets/LSG/manuals/trizol_reagent.pdf

4 https://assets.thermofisher.com/TFS-Assets/LSG/manuals/superscriptIII_man.pdf

## References

[1] Zhiguo E, Wang L, Zhou J (2013). Splicing and alternative splicing in rice and humans. BMB Rep.

[2] Wang Y, Liu J, Huang B, Xu Y-M, Li J, Huang L-F, Lin J, Zhang J, Min Q-H, Yang W-M (2015). Mechanism of alternative splicing and its regulation. Biomed Rep.

[3] Zhang W, Chang J-W, Lin L, Minn K, Wu B, Chien J, Yong J, Zheng H, Kuang R (2015). Network-based isoform quantification with RNA-seq data for cancer transcriptome analysis. PLoS Comput Biol.

[4] Sun J, Chang J, Zhang T, Yong J, Kuang R, Zhang W. Platform-integrated mRNA Isoform Quantification. Bioinformatics (Oxford, England). 2019. 10.1093/bioinformatics/btz932. 10.1093/bioinformatics/btz932 PMC7178424 31834359

[5] Dapas M, Kandpal M, Bi Y, Davuluri RV (2016). Comparative evaluation of isoform-level gene expression estimation algorithms for RNA-seq and exon-array platforms. Brief Bioinforma.

[6] Zhao S, Fung-Leung W-P, Bittner A, Ngo K, Liu X (2014). Comparison of RNA-Seq and microarray in transcriptome profiling of activated T cells. PLoS ONE.

[7] Wang Z, Gerstein M, Snyder M (2009). RNA-Seq: a revolutionary tool for transcriptomics. Nat Rev Genet.

[8] Geiss GK, Bumgarner RE, Birditt B, Dahl T, Dowidar N, Dunaway DL, Fell HP, Ferree S, George RD, Grogan T (2008). Direct multiplexed measurement of gene expression with color-coded probe pairs. Nat Biotechnol.

[9] Kulkarni MM. Digital multiplexed gene expression analysis using the NanoString nCounter system. Curr Protocol Mol Biol. 2011:25–10. 10.1002/0471142727.mb25b10s94. 10.1002/0471142727.mb25b10s94 21472696

[10] Barretina J, Caponigro G, Stransky N, Venkatesan K, Margolin AA, Kim S, Wilson CJ, Lehár J, Kryukov GV, Sonkin D (2012). The Cancer Cell Line Encyclopedia enables predictive modelling of anticancer drug sensitivity. Nature.

[11] Edgar R, Domrachev M, Lash AE (2002). Gene Expression Omnibus: NCBI gene expression and hybridization array data repository. Nucleic Acids Res.

[12] Guo Y, Sheng Q, Li J, Ye F, Samuels DC, Shyr Y (2013). Large scale comparison of gene expression levels by microarrays and RNAseq using TCGA data. PLoS ONE.

[13] Wang C, Gong B, Bushel PR, Thierry-Mieg J, Thierry-Mieg D, Xu J, Fang H, Hong H, Shen J, Su Z (2014). The concordance between RNA-seq and microarray data depends on chemical treatment and transcript abundance. Nat Biotechnol.

[14] Futreal PA, Coin L, Marshall M, Down T, Hubbard T, Wooster R, Rahman N, Stratton MR (2004). A census of human cancer genes. Nat Rev Cancer.

[15] Trapnell C, Williams BA, Pertea G, Mortazavi A, Kwan G, Van Baren MJ, Salzberg SL, Wold BJ, Pachter L (2010). Transcript assembly and quantification by RNA-Seq reveals unannotated transcripts and isoform switching during cell differentiation. Nat Biotechnol.

[16] Li B, Dewey CN (2011). RSEM: accurate transcript quantification from RNA-Seq data with or without a reference genome. BMC Bioinformatics.

[17] Roberts A, Pachter L (2013). Streaming fragment assignment for real-time analysis of sequencing experiments. Nat Methods.

[18] Bray NL, Pimentel H, Melsted P, Pachter L (2016). Near-optimal probabilistic rna-seq quantification. Nat Biotechnol.

[19] Dobin A, Davis CA, Schlesinger F, Drenkow J, Zaleski C, Jha S, Batut P, Chaisson M, Gingeras TR (2013). STAR: ultrafast universal RNA-seq aligner. Bioinformatics.

[20] O’Leary NA, Wright MW, Brister JR, Ciufo S, Haddad D, McVeigh R, Rajput B, Robbertse B, Smith-White B, Ako-Adjei D (2015). Reference sequence (refseq) database at ncbi: current status, taxonomic expansion, and functional annotation. Nucleic Acids Res.

[21] Turro E, Lewin A, Rose A, Dallman MJ, Richardson S (2009). MMBGX: a method for estimating expression at the isoform level and detecting differential splicing using whole-transcript Affymetrix arrays. Nucleic Acids Res.

[22] Irizarry RA, Hobbs B, Collin F, Beazer-Barclay YD, Antonellis KJ, Scherf U, Speed TP (2003). Exploration, normalization, and summaries of high density oligonucleotide array probe level data. Biostatistics.

[23] Anderson MJ (2001). A new method for non-parametric multivariate analysis of variance. Austral Ecol.

