# Supplementary material for: A large-scale comparative study of isoform expressions measured on four platforms
Source: BMC Genomics. 2020 Mar 30;21:272. doi: 10.1186/s12864-020-6643-8 (PMC7106849; doi:10.1186/s12864-020-6643-8)
Supplement: Supplementary file 1 — Additional file 1 Figures S1-S6 and Table S1-S2. [file 12864_2020_6643_MOESM1_ESM.pdf]

Supplementary

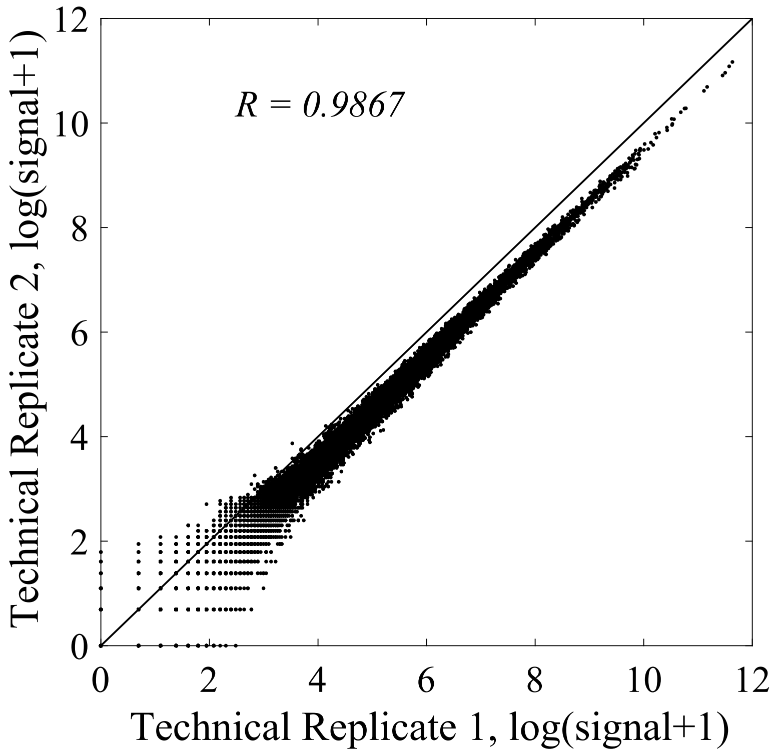

Figure S1: **Reproducibility.** NanoString correlation plot showing technical replicates of 59 cell line samples. Normalized log values of signal are shown.

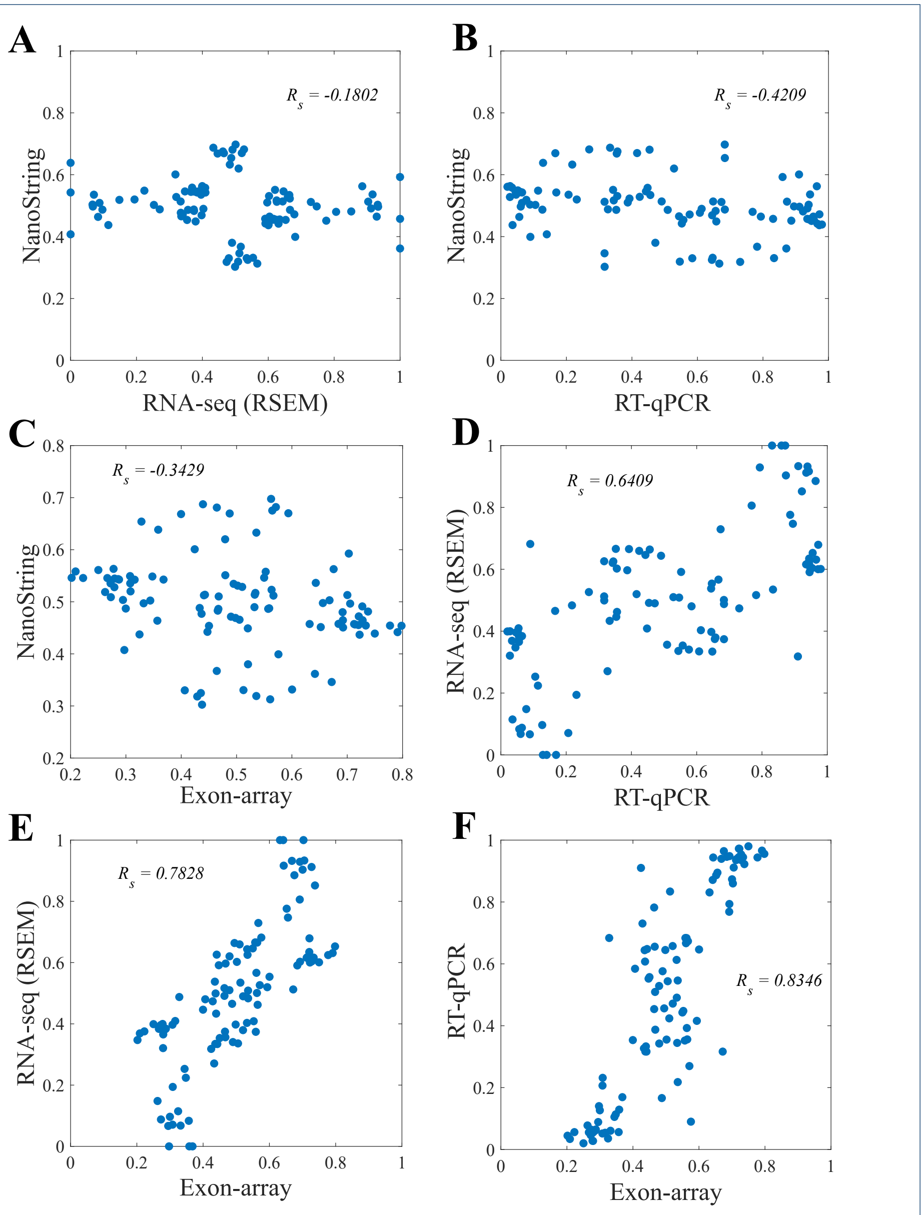

Figure S2: **Scatter plots of isoform proportions estimated on two platforms.** Each dot represents one isoform in one cell line. RSEM is applied for RNA-seq analysis.

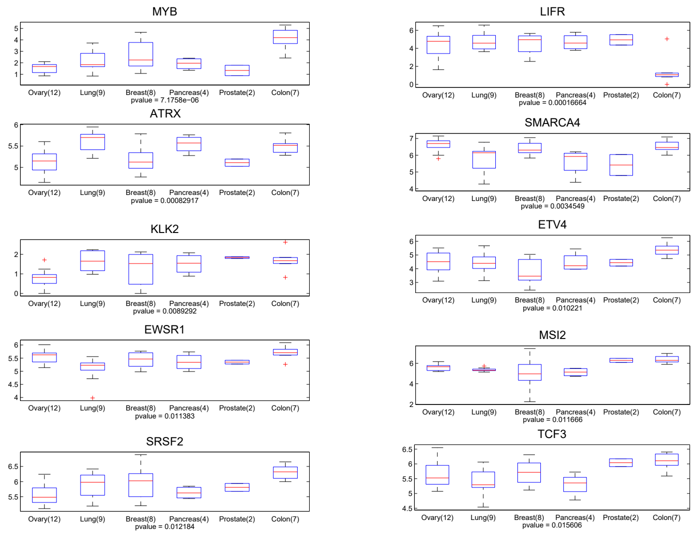

Figure S3: **The Top 10 genes identified from NanoString platform by ANOVA.** The y-axis shows the expression levels. The numbers in the parentheses represent the number of cell lines in each tissue type.

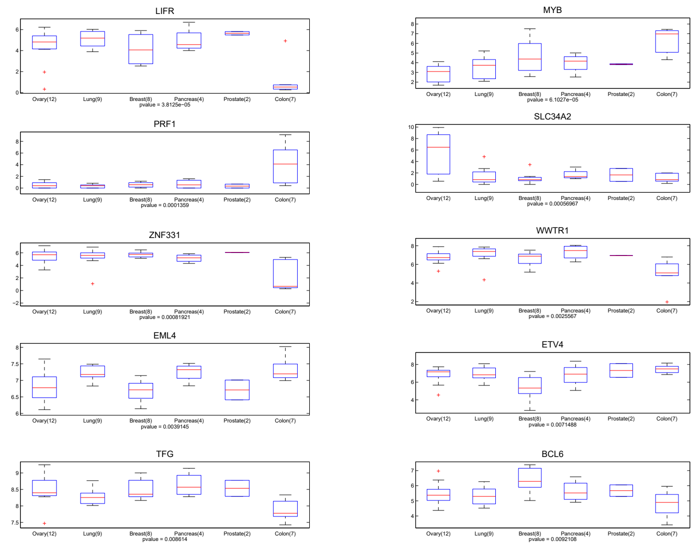

Figure S4: **The Top 10 genes identified from RNA-seq platform by ANOVA.** The y-axis shows the expression levels. The numbers in the parentheses represent the number of cell lines in each tissue type.

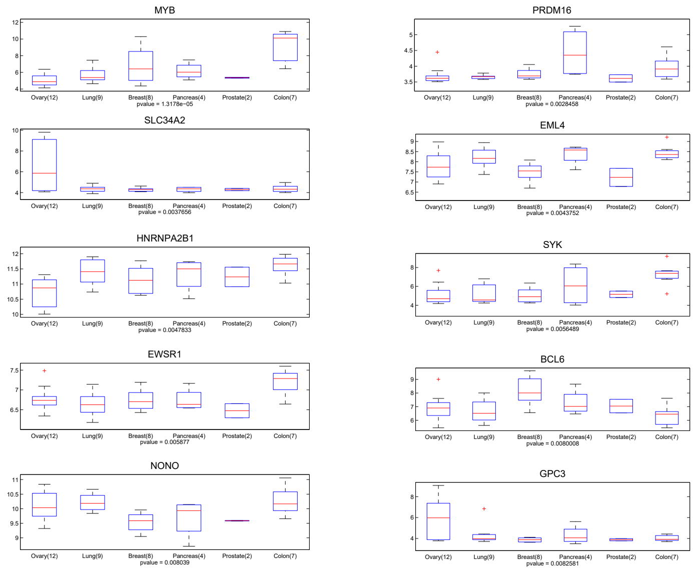

Figure S5: **The Top 10 genes identified from Microarray platform by ANOVA.** The y-axis shows the expression levels. The numbers in the parentheses represent the number of cell lines in each tissue type.

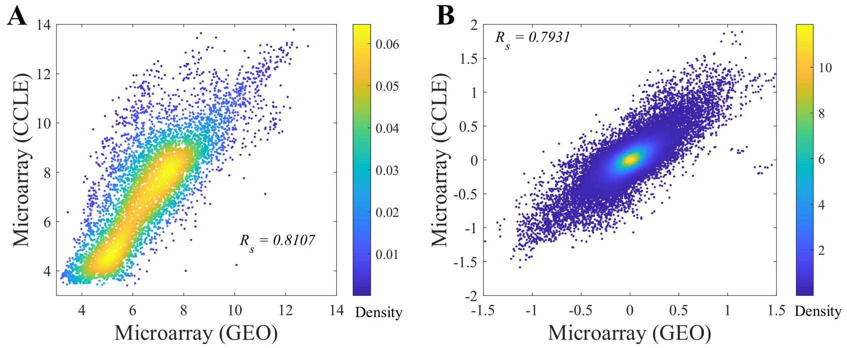

Figure S6: **(A) Scatter plot of gene expressions estimated by Microarray CCLE and GEO. (B) Scatter plot of fold-changes in gene expressions (breast cancer cell line versus another cell line) between Microarray CCLE and GEO.**

**Source & Cell Line Name**		Catalogue Number	NanoString	RNA-seq (CCLE)	Microarray		Exon-array (GEO)
					CCLE	GEO	
Ovary	NIH:OVCAR-3	ATCC HTB-161	1	1	1	GSM803658	GSM736089
	A2780	SIGMA 93112519	1	1	1	GSM1374381	GSM1291129
	Hey-A8	RRID CVCL_8878	1	1	1	GSM529586	NA
	SK-OV-3	ATCC HTB-77	1	1	1	GSM803662	GSM736094
	RMG-I	RRID CVCL_1662	1	1	1	GSM887544	NA
	OVCAR-4	RRID CVCL_1627	1	1	1	GSM803659	GSM736090
	Caov-3	ATCC HTB-75	1	1	1	GSM274705	GSM1291130
	OVSAHO	RRID CVCL_3114	1	1	1	GSM1230854	NA
	ES-2	ATCC CRL-1978	1	1	1	GSM274699	GSM1291140
	TOV-21G	ATCC CRL-11730	1	1	1	GSM1230710	GSM1291153
	KURAMOCHI	RRID CVCL_1345	1	1	1	GSM1230827	NA
	OVCAR-8	RRID CVCL_1629	1	1	1	GSM803661	GSM736092
Lung	DMS53	ATCC CRL-2062	1	1	1	GSM1060792	NA
	NCI-H1299	ATCC CRL-5803	1	1	1	GSM274743	NA
	NCI-H460	ATCC HTB-177	1	1	1	GSM803655	GSM736074
	Calu-1	ATCC HTB-54	1	1	1	GSM274735	NA
	Calu-3	ATCC HTB-55	1	1	1	GSM357899	NA
	SK-MES-1	ATCC HTB-58	1	1	1	GSM274789	NA
	A549	ATCC CCL-185	1	1	1	GSM803648	GSM736067
	NCI-H358	ATCC CRL-5807	1	1	1	GSM274731	NA
	HCC-H827	ATCC CRL-2868	1	1	1	GSM183436	NA
	MRC-5	ATCC CCL-171	1	NA	NA	NA	NA
	IMR90	ATCC CCL-186	1	NA	NA	NA	GSM541279
	WI38	ATCC CCL-75	1	NA	NA	NA	GSM651580
Colon	HCT116	ATCC CCL-247	1	1	1	GSM803635	GSM736062
	SW480	ATCC CCL-228	1	1	1	GSM274771	NA
	DLD-1	ATCC CCL-221	1	NA	NA	NA	GSM1132671
	HT-29	ATCC HTB-38	1	1	1	GSM803637	GSM736064
	KM12C	RRID CVCL_9547	1	1	1	GSM803638	GSM736065
	KM12SM	RRID CVCL_9548	1	NA	NA	NA	NA
	HCT-15	ATCC CCL-225	1	1	1	GSM803636	GSM736063
	SW620	ATCC CCL-227	1	1	1	GSM803639	GSM736066
	LS-174T	ATCC CL-188	1	NA	NA	NA	NA
	Caco-2	ATCC HTB-37	1	NA	NA	NA	GSM472933
	Lovo	ATCC CCL-229	1	1	1	GSM274719	NA
Breast	MCF-7	ATCC HTB-22	1	1	1	GSM803623	GSM419264
	MCF 10A	ATCC CRL-10317	1	NA	NA	NA	GSM1098809
	BT-549	ATCC HTB-122	1	1	1	GSM803621	GSM419258
	MDA-MB-231	ATCC HTB-26	1	1	1	GSM803625	GSM419268
	T47D	ATCC HTB-133	1	1	1	GSM803673	GSM419291
	SK-BR-3	ATCC HTB-30	1	1	1	GSM320611	GSM419279
	Hs578T	ATCC HTB-126	1	1	1	GSM803622	GSM419263
	MDA-MB-436	ATCC HTB-130	1	1	1	GSM320608	GSM419273
	HCC1937	ATCC CRL-2336	1	1	1	GSM320621	GSM419262
	HCC1937/BRCA1	ATCC CRL-2336	1	NA	NA	NA	NA
Pancreas	Capan-1	ATCC HTB-79	1	1	1	GSM1374439	NA
	MIA-Paca2	ATCC CRL-1420	1	1	1	GSM450325	NA
	PANC-1	ATCC CRL-1469	1	1	1	GSM1374808	GSM472938
	BxPC-3	ATCC CRL-1687	1	1	1	GSM1374417	NA
Prostate	DU145	ATCC HTB-81	1	1	1	GSM803663	GSM736095
	PC-3	ATCC CRL-1435	1	1	1	GSM803664	GSM736096
Stomach	AGS	ATCC CRL-1739	1	1	1	GSM552354	GSM831348
	SNU484	RRID CVCL_0100	1	NA	NA	NA	NA
Urinary bladder	J82	ATCC HTB-1	1	1	1	GSM1374576	NA
Cervix	Hela	ATCC CCL-2	1	NA	NA	NA	GSM772580
Connective tissue	HT-1080	ATCC CCL-121	1	1	1	GSM253204	GSM969711
Liver	HepG2	ATCC HB-8065	1	1	1	GSM253212	GSM472906
Skin	A431	ATCC CRL-1555	1	NA	NA	NA	NA
Epithelial keratinocyte	Hacat	RRID CVCL_0038	1	NA	NA	NA	NA
**Total**			**59**	**46**	**46**	**46**	**35**

Table S1: Cell lines with different platforms in the experiments.

NanoString		RNA-seq		Microarray	
Gene Name	p-value	Gene Name	p-value	Gene Name	p-value
MYB	7.18E-06	LIFR	3.81E-05	MYB	1.32E-05
LIFR	0.0001666384	MYB	6.10E-05	PRDM16	0.0028458423
ATRX	0.0008291736	PRF1	0.0001359048	SLC34A2	0.0037655683
SMARCA4	0.0034549266	SLC34A2	0.0005696696	EML4	0.0043751938
KLK2	0.0089292042	ZNF331	0.0008192107	HNRNPA2B1	0.0047833351
ETV4	0.0102211872	WWTR1	0.0025567496	SYK	0.0056488715
EWSR1	0.0113829476	EML4	0.0039144826	EWSR1	0.0058769717
MSI2	0.0116660102	ETV4	0.0071488179	BCL6	0.0080007656
SRSF2	0.0121842143	TFG	0.0086139687	NONO	0.0080389793
TCF3	0.0156059695	BCL6	0.0092107745	GPC3	0.0082581154
LCK	0.0166432818	CBFB	0.0103664567	PPARG	0.008873531
TFG	0.0180887349	TCF12	0.0106408736	WWTR1	0.009058997
CCNB1IP1	0.0183982375	TFRC	0.0156299922	NUP98	0.0096644936
LMO2	0.0213889791	C2orf44	0.0174459151	TCF12	0.0112737393
MYCL1	0.0221279408	PRDM16	0.017847213	DICER1	0.0119999628
NFKB2	0.0292696619	BRCA1	0.0180211927	PRF1	0.0138773905
SS18	0.0312757309	NIN	0.0183256485	CREB3L2	0.014550245
VHL	0.0366680532	CREB3L2	0.0193301112	BRCA1	0.0179519204
FBXO11	0.0369399304	SMARCB1	0.0213776167	LCK	0.0200464659
NR4A3	0.0374723173	NOTCH2	0.0264918821	NKX2-1	0.0215930341
SYK	0.0403151922	SS18	0.0289741933	NIN	0.0259918914
BIRC3	0.0427168466	IL6ST	0.0387334796	CTNNB1	0.0303476612
TFRC	0.0450303995	PPARG	0.0409239462	PBX1	0.0320564792
APC	0.0462791202	LCK	0.0414188322	FANCA	0.0322131111
EPS15	0.0475854976	CDKN2C	0.0441469827	SS18	0.0342815245
		MSI2	0.0451392002	NSD1	0.0342827581
		PRDM1	0.0493855961	ZNF331	0.0353209906
				LIFR	0.0368468569
				NOTCH2	0.0376660514
				BIRC3	0.0412805592
				ETV4	0.0413177537
				CBFA2T3	0.0426270441
				TFG	0.0485835517
				MLLT10	0.0488134998

Table S2: The differentially expressed genes (ANOVA p-value < 0.05). The genes identified by three platforms are marked in red and by two platforms are marked in blue.
